# β-Catenin determines upper airway progenitor cell fate and preinvasive squamous lung cancer progression by modulating epithelial–mesenchymal transition

**DOI:** 10.1002/path.3962

**Published:** 2012-01-17

**Authors:** Adam Giangreco, Liwen Lu, Charles Vickers, Vitor Hugo Teixeira, Karen R Groot, Colin R Butler, Ekaterina V Ilieva, P Jeremy George, Andrew G Nicholson, Elizabeth K Sage, Fiona M Watt, Sam M Janes

**Affiliations:** 1 Centre for Respiratory Research, University College LondonUK; 2 Department of Thoracic Medicine, University College London HospitalUK; 3 Department of Histopathology, Royal Brompton HospitalLondon, UK; 4 Epithelial Cell Biology Laboratory, Cancer Research UK Cambridge Research InstituteUK

**Keywords:** β-catenin, E-cadherin, Snail, airway, stem cell, pre-invasive, lung cancer, squamous

## Abstract

Human lung cancers, including squamous cell carcinoma (SCC) are a leading cause of death and, whilst evidence suggests that basal stem cells drive SCC initiation and progression, the mechanisms regulating these processes remain unknown. In this study we show that β-catenin signalling regulates basal progenitor cell fate and subsequent SCC progression. In a cohort of preinvasive SCCs we established that elevated basal cell β-catenin signalling is positively associated with increased disease severity, epithelial proliferation and reduced intercellular adhesiveness. We demonstrate that transgene-mediated β-catenin inhibition within keratin 14-expressing basal cells delayed normal airway repair while basal cell-specific β-catenin activation increased cell proliferation, directed differentiation and promoted elements of early epithelial-mesenchymal transition (EMT), including increased Snail transcription and reduced E-cadherin expression. These observations are recapitulated in normal human bronchial epithelial cells *in vitro* following both pharmacological β-catenin activation and E-cadherin inhibition, and mirrored our findings in preinvasive SCCs. Overall, the data show that airway basal cell β-catenin determines cell fate and its mis-expression is associated with the development of human lung cancer. Copyright © 2012 Pathological Society of Great Britain and Ireland. Published by John Wiley & Sons, Ltd.

## Introduction

Lung cancers including squamous cell carcinomas (SCCs) are a leading cause of death and carry a poor prognosis with the majority of patients presenting at a stage when curative treatments are no longer feasible. SCCs are characterized by abundant keratin protein expression and exhibit stereotypical progression from normal tissue through preinvasive metaplasia, dysplasia, carcinoma *in situ*, and eventual invasive carcinoma [1–3]. Progressive changes in lung and other cancers include loss of intercellular adhesions, acquisition of unlimited self-renewal capacity and increased expression of mesenchymal cell characteristics; these are known collectively as epithelial–mesenchymal transition (EMT) [4]. Although several techniques can now detect preinvasive lung cancers within large airways, evidence indicates that only half of these eventually progress to invasive carcinoma [5]. An improved understanding of the cellular and molecular pathways that determine lung cancer progression is therefore required.

Evidence now points to a functional role for keratin 14 (K14)-expressing basal cells in the initiation, progression and severity of human SCCs. Specifically, it has been determined that these cells act as unique SCC tumour-initiating cells [1, 2] and that keratin 14 gene enrichment within SCCs is independently associated with poor patient prognosis [1]. Transgene lineage–tracing studies carried out using both keratin 5 (K5) and K14 basal cell-specific promoters indicate that subsets of tracheal basal cells function as stem cells in mammalian airways. Specifically, genetic recombination within basal cells can identify stable, self-renewing cell clones that exhibit both injury-dependent expansion and multipotent cell differentiation [6–8]. Lineage-labelled cells display robust *in vitro* and *in vivo* growth capacity and can recapitulate all of the differentiated cell lineages present within proximal airways [6, 8–12].

Recent gene expression studies have determined that basal cells maintain elevated Wnt/β-catenin activity relative to other tracheal epithelial cells [8]. This pathway is a known regulator of distal lung development, differentiation and bronchiolar stem cell abundance [13–17]. Transgene-mediated β-catenin activation can also enhance distal lung repair and increases the clonogenic potential of bronchiolar stem cells [13]. In other tissues β-catenin is known to promote transcription of downstream target genes, including Twist, Snail, Slug and matrix metalloproteinases. These genes influence both intercellular and cell-matrix adhesiveness and provide a mechanism through which β-catenin regulates EMT phenotypes in many normal and cancerous tissues [18].

In this study we test the hypothesis that β-catenin determines cell phenotype in normal and precancerous mammalian airway basal cells. We show that elevated β-catenin signalling within human preinvasive lung cancers is associated with increased disease severity, epithelial proliferation and the promotion of an early EMT phenotype. We demonstrate in murine models that basal cell-specific β-catenin activation is both necessary and sufficient for tracheal growth, differentiation and homeostasis and that β-catenin accomplishes these effects via regulation of cadherin-dependent adhesion. Our study therefore provides novel links between lung stem cell β-catenin signalling, cell adhesiveness and lung cancer ontogeny.

## Materials and methods

### Mouse husbandry

Adult, 2–6 month-old mice were used for experiments, housed in individually ventilated cages on a 12 h light–dark cycle and allowed access to food and water *ad libitum*. Transgenic K14-dnLef1 and K14-Δβ-catenin ER mice were provided by Professor Fiona Watt and generated as previously described [19–21]. TOPgal transgenic mice were obtained from a commercial vendor and generated as previously described [22] (Jackson Laboratories, ME, USA). All transgenic mice were maintained on a mixed background; the D4 mice were a mix of 129 and C57/Bl6, the TOPgal mice were CD1 background. Transgene-negative, age and sex-matched littermates were used as controls. For experiments involving tracheal repair, the mice were anaesthetized with isofluorane and the tracheas damaged via oropharyngeal instillation of 15 µl 2% polidocanol (Sigma, UK). The mice were sacrificed by sodium pentobarbitol overdose and sentinel screenings for common murine pathogens were used throughout the course of these studies. All *in vivo* experiments involved a minimum sample size of five animals/group, were repeated at least twice and were performed under the terms of a UK Home Office project license and the in-house animal ethics committee.

### Flow cytometry

Single-cell suspensions were derived from mouse tracheas after overnight 0.1% pronase digestion at 4 °C and stained for epithelial and immune cell markers, including CD45, EpCam and GSI-β4 lectin, as previously described [8, 12, 23]. Dead cells were excluded on the basis of DAPI reactivity and epithelial and basal cells identified based on cell surface epithelial cell adhesion molecule (EpCam) and lectin subunit from *Griffonia simplicifolia* (GSI-β4) lectin expression. TOPgal activity was detected by fluorescein di-β-D-galactopyranoside (FDG) staining, as previously described [24]. Flow cytometric cell cycle analysis of human bronchial epithelial cells was also performed, as previously described [25]. All data were obtained using an LSR II flow cytometer (BD Biosciences). Single-stained controls were used to set compensation parameters and unstained samples used to set negative gates.

### *In vitro* experiments

Tracheas were harvested using aseptic techniques, pooled, cleaned, digested in pronase and cultured as previously described [10]. Enzyme isolated cells were counted and seeded in 12-well Transwell dishes (Corning) at 2 × 10^5^ cells/well in MTEC/plus. Upon visually reaching confluence (day 7 post-plating), the medium was changed to MTEC/basic plus retinoic acid and cells were subsequently cultured at an air–liquid interface [10]. Immortalized human bronchial epithelial cells were obtained from Gisli Jenkins (Nottingham, UK). SB415286 was administered for a total of 24 h to all iHBEC cultures at a dose of 30 µM.

### Tissue preparation, histology and antibody staining

Human and murine tissue sections were fixed overnight in 10% neutral buffered formalin or 4% paraformaldehyde, processed and sectioned at 5 µm thickness. Human biopsy samples were obtained via fibre-optic bronchoscopy with full patient consent under relevant local and national authority ethical approval (REC Approval No. 06/Q0505/12). Human and murine haematoxylin and eosin (H&E) staining was performed using an automated staining system (TissueTek). All human biopsy specimens were subjected to routine histopathological diagnosis and a further histopathologist provided complete agreement on diagnosis. In some cases, fresh murine TOPgal transgenic tracheas were dissected longitudinally, fixed in 4% paraformaldehyde for 1 h, washed extensively in PBS, incubated overnight in X-gal staining solution at 37 °C and post-fixed in 4% paraformaldehyde, as previously described [26]. Air–liquid interface (ALI) culture wholemounts were fixed overnight in 4% paraformaldehyde prior to antibody immunostaining.

Immunofluorescent or immunohistochemical human and murine tissue section and/or ALI wholemount antibody staining followed standard conditions [27]. Primary antibodies included keratin 14 (rabbit; ABR Biosystems), CCSP (goat; provided by Dr Barry Stripp), BrdU (sheep; Abcam), acetylated tubulin (mouse; Sigma), ZO-1 (rabbit; Sigma), E-cadherin (rat; Zymed) and Cyclin D1 (rabbit; Abcam). Species-appropriate secondary antibodies included streptavidin–HRP (β-catenin, Ki67) or directly conjugated AlexaFluor dyes (all other primary antibodies). Images were acquired using a Leica TCS Tandem or SPE confocal or an Olympus brightfield microscope. All images were post-processed using Adobe Photoshop (Photomerge, rotate, crop, brightness and contrast adjustments).

### Morphometry and statistical analysis

Epithelial height and cell density were determined using Volocity image-analysis software. We quantitated the abundance of specific tracheal cell phenotypes as a function of epithelial basement membrane length [28, 29]. Cell surface E-cadherin abundance was quantitated as previously described [30]. β-Catenin staining of epithelial cells was quantified as previously described [31].

Pairwise *t*-tests with Welch's correction to account for unequal sample variance were used for all statistical comparisons. Statistical significance was accepted at *p* < 0.05 for all analyses. Graphs are presented as box-and-whisker plots, in which the box represents standard error of the mean (SEM) and whiskers any outlier populations. All statistical analyses were performed using GraphPad Prism and Microsoft Excel.

### Western blot analysis

Cells isolated from *in vitro* cultures or whole tracheal samples were washed briefly, frozen in liquid nitrogen and lysed in RIPA buffer supplemented with complete protease inhibitors (Roche) and phosSTOP phosphatase inhibitor (Roche). Equal amounts of protein were analysed by western blotting, using antibodies against phospho-Gsk3β, acetylated tubulin, Cyclin D1 and β-tubulin (Sigma).

### Quantitative RT–PCR

Total RNA was isolated from cultured or freshly isolated human and murine tracheal epithelial cells using a commercially available SV RNA isolation kit (Promega). Quantitative PCR was performed under standard conditions using an Eppendorf real-time PCR machine. All samples were run as triplicates with a minimum of two samples/treatment type. Relative RNA quantitation was based on 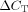 calculations, and all samples were compared with each other using β2-microglobulin and 18S as controls. Taqman predesigned, recommended, inventoried probes and 2× PCR Master Mix (Applied Biosystems) were used for all analyses.

## Results

### Enhanced β-catenin signalling is associated with increased lung cancer severity

**Figure 1:**
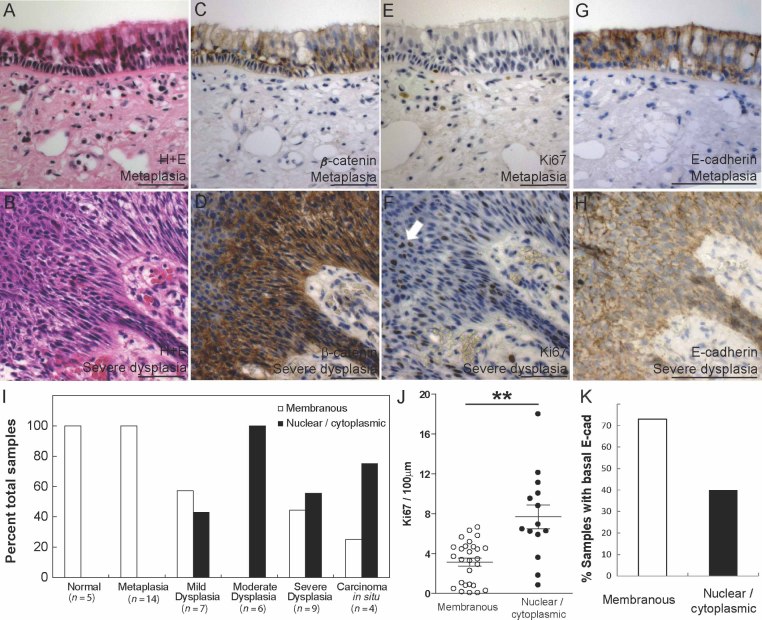
β-Catenin signalling is positively associated with human preinvasive lung cancer severity. (A–H) Representative images of adjacent serial sections from metaplastic (A, C, E, G) and severely dysplastic (B, D, F, H) human lung cancer biopsies stained with H
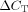
E (A, B) or antibodies directed against β-catenin (C, D), Ki67 (E, F), and E-cadherin (G, H). (I) Quantitation of the percentage of biopsy samples exhibiting membranous (white bars) or nuclear/cytoplasmic β-catenin (black bars) in the tissue section per disease stage. (J, K) Ki67 incidence and basal E-cadherin quantitation in samples exhibiting membranous or nuclear/cytoplasmic β-catenin (white and black bars, respectively). Scale bars = 100 µm; *p < 0.005

To ascertain whether β-catenin signalling was associated with preinvasive lung cancer severity, we examined intracellular β-catenin localization in 45 biopsy samples from patients with various stages of preinvasive SCC (normal, metaplasia, mild, moderate and severe dysplasia, carcinoma *in situ*). H&E staining revealed dramatic differences in tissue morphology between each disease stage (cf metaplasia and severe dysplasia, Figure 1A, b). All normal and metaplastic lung tissue sections stained for β-catenin, exhibited robust membrane-associated β-catenin and no nuclear or cytoplasmic protein localization (Figure 1C). In contrast, samples with mild to severe dysplasia or carcinoma *in situ* frequently exhibited nuclear and cytoplasmic β-catenin protein abundance (Figure 1D; data quantitated in Figure 1I).

We used Ki67 and E-cadherin immunostaining to determine whether proliferation or intercellular adhesiveness might be altered in conjunction with β-catenin signalling. Cell proliferation was increased in samples exhibiting nuclear or cytoplasmic β-catenin localization relative to those with membrane-associated β-catenin (Figure 1E, f). In severely dysplastic and carcinoma *in situ* samples with nuclear or cytoplasmic β-catenin expression, cell proliferation was frequently not restricted to the epithelial basement membrane (arrow, Figure 1F). E-cadherin abundance was also reduced in basal cells with nuclear or cytoplasmic β-catenin localization (Figure 1G, h). Thus, nuclear or cytoplasmic β-catenin localization was positively associated with both increased proliferation and reduced E-cadherin abundance (Figure 1J, k).

### Tracheal basal cells activate canonical Wnt signalling during repair

**Figure 2:**
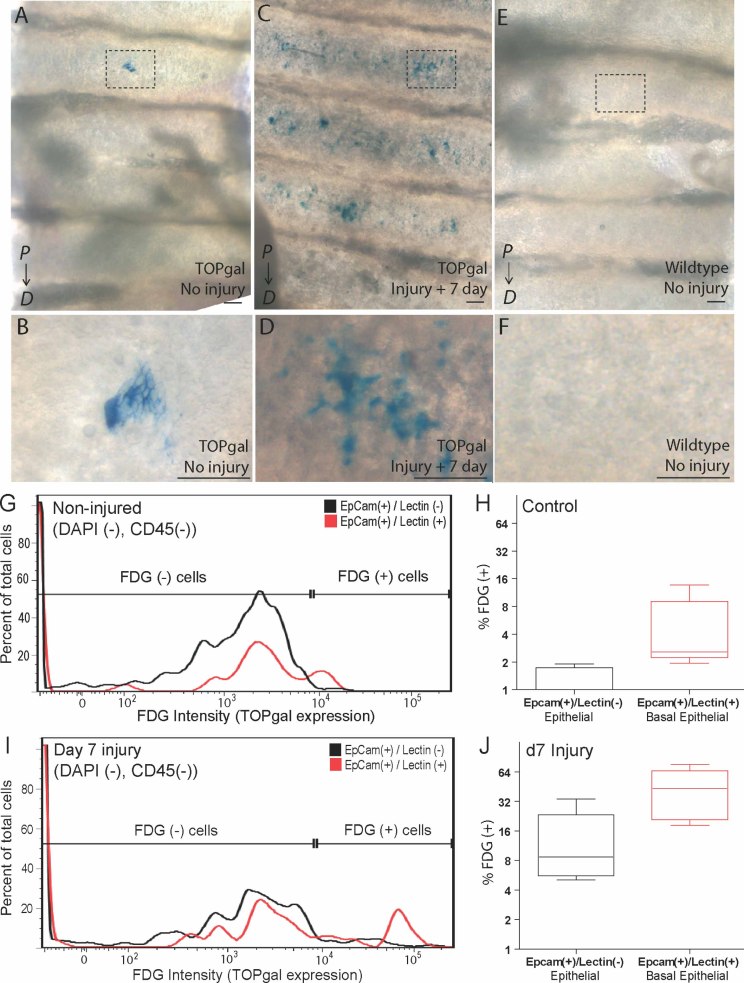
Tracheal epithelial repair is associated with basal cell β-catenin activation. (A–F) Whole mounts of TOPgal (A–D) or wild-type (E, F) uninjured (A, B, E, F) and polidocanol-damaged (C, D) tracheas stained using X-gal to detect β-catenin activity (blue). Dashed boxes (A, C, E) indicate areas magnified in (B, D, F). (G–J) Flow cytometry of undamaged TOPgal control (G, H) or 7 day recovered TOPgal tracheal epithelial cells (I, J). Cells isolated from tracheas were labelled using DAPI, CD45, EpCam, GSI-β4 lectin and FDG (see Supporting information, Figure S1). FDG-reactive, non-haematopoietic EpCam^+^/lectin^−^ (non-basal, black, G–J) and EpCam^+^/lectin^+^ (basal, red, G–J) epithelial cell abundance was determined for control (G, H) and polidocanol damaged samples (I, J). (H, J) Box-with-whisker plots, with the box being SEM and whiskers denoting outliers. The tracheal proximal–distal (P–D) axis is indicated (A, C, E); scale bars = 100 µm

To assess canonical Wnt (β-catenin–TCF signalling) in normal and repairing airways we used TOPgal β-catenin reporter mice [22]. Mice were untreated or given 2% polidocanol by oropharyngeal instillation to damage their tracheas. Oropharyngeal polidocanol causes epithelial cell desquamation within 24 h, followed by robust basal cell proliferation within 48–72 h and restoration of a differentiating epithelium 7–10 days post-damage [32]. Normal and polidocanol-damaged tracheas recovered for 7 days were stained with X-gal to detect β-galactosidase reporter gene activity [22]. There was only sporadic reporter gene activity in two of four tracheas in the absence of damage; this was restricted to small clusters of 10–20 cells (blue-stained cells, Figure 2A, b). After damage, all samples exhibited robust reporter gene activity throughout the trachea (Figure 2C, D). X-gal staining was never detected in transgene-negative (wild-type) controls (Figure 2E, f). These results were consistent with previous findings in distal airways and demonstrate that β-catenin–TCF signalling is associated with tracheal epithelial repair [13].

To determine the abundance and phenotype of tracheal β-catenin–TCF signalling cells, we used flow cytometry and FDG, EpCam and GSIβ4 lectin antibody staining (to detect TOPgal expression, epithelial and basal cell types, respectively; Figure 2G–J). Freshly isolated cells were selected based on forward and side scatter, stained with DAPI and CD45 and gated as shown in Figure S1 (see Supporting information). Non-transgenic mice and single-stain controls were used to set all gates. In the absence of injury, only rare EpCam^+^/GSI-β4 lectin^+^ basal cells exhibited FDG staining resulting from TOPgal gene activity (red lines and bars, Figure 2G, h). In contrast, FDG staining was present in 20–60% of EpCam^+^/lectin^+^ basal cells and 5–20% of non-basal EpCam^+^/lectin^−^ cells 7 days after damage (Figure 2I, j). To assess whether this TOPgal transgene activation was associated with increased cell proliferation, we also used FDG labelling and flow cytometry to assess the cell cycle in combination with GSI-β4 lectin^+^. FDG-positive basal cells had significantly increased proliferation relative to FDG-negative basal cells (see Supporting information, Figure S2). Interestingly, non-basal FDG-positive cells did not exhibit increased proliferation relative to FDG-negative non-basal cells (see Supporting information, Figure S2D). These results demonstrate that tracheal β-catenin–TCF signalling occurs predominantly within basal cells and is associated in these cells with increased proliferation.

### Stem cell β-catenin–TCF signalling is necessary for normal tracheal repair

**Figure 3:**
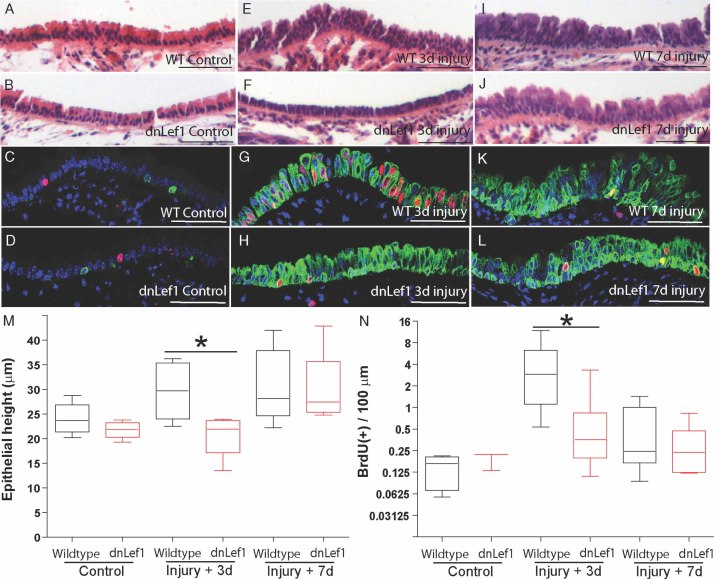
Basal cell β-catenin inhibition delays normal epithelial repair. (A–L) H&E staining (A, B, E, F, I, J) and indirect immunofluorescence (C, D, G, H, K, L) using keratin 14 (green) plus BrdU (red) antibodies in uninjured (A–D) or polidocanol injured, 3 (E–H) and 7 day (I–L) recovered wild-type (A, E, I, C, G, K) and K14-dnLef1 transgenic tracheas (B, F, J, D, H, L). (M) Quantitation of average epithelial height in uninjured control and polidocanol damaged wild-type and K14-dnLef1 tracheas (black and red bars, respectively). (N) Quantitation of BrdU-reactive cell incidence/100 µm basement membrane length in control, 3 day and 7 day recovered wild-type and transgenic tracheas (black and red bars). Plots (M, N) are box-with-whisker; scale bars = 100 µm; *p < 0.05

We investigated whether basal cell canonical Wnt–β-catenin signalling was necessary for tracheal homeostasis or repair, using a K14-dominant negative (*N*-terminally truncated) Lef1 transgenic mouse model (K14-dnLef1 [21]). H&E staining and indirect antibody immunofluorescence revealed no differences between uninjured wild-type and K14-dnLef1 tracheal morphology, basal progenitor cell abundance and epithelial proliferation (Figure 3A–D; quantitation in Figure 3M, n). Additionally, although it has been previously reported that Lef1 was required for tracheal submucosal gland development [33, 34], both K14-dnLef1 and wild-type tracheas exhibited comparable submucosal gland abundance, distribution and cellular phenotypes (see Supporting information, Figure S3). This agreed with previous reports that β-catenin–TCF signalling is dispensable for airway homeostasis [26].

To assess whether tracheal repair was perturbed in the absence of basal cell β-catenin signalling, we compared polidocanol-damaged wild-type and K14-dnLef1 transgenic tracheas. Three days after damage, the epithelial height and cell density of K14-dnLef1 transgenic tracheas was reduced compared with wild-type (Figure 3E, f; quantitated in Figure 3M). Although this difference was not dependent on reduced K14-dnLef1 basal cell abundance, we did observe significantly reduced cell proliferation in transgenic mice recovered for 3 days (Figure 3G, h, n). Seven days after damage, cell proliferation, epithelial height and cellular phenotype were all similar between wild-type and transgenic mice. Thus, stem cell β-catenin signalling is necessary for early tracheal repair.

### β-Catenin–TCF signalling increases basal cell proliferation and determines epithelial differentiation

**Figure 4:**
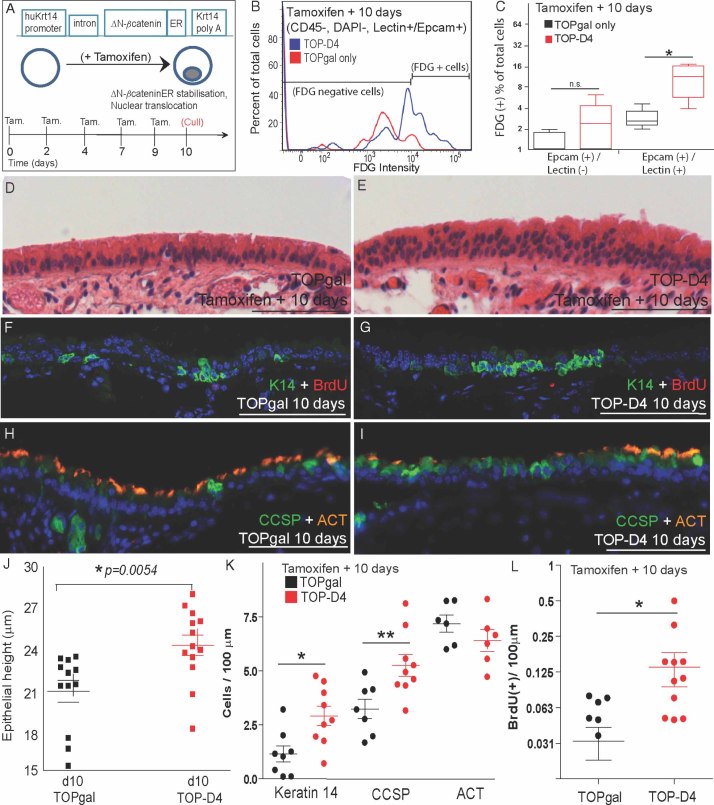
Basal cell β-catenin activity determines tracheal growth and lineage choice. (A) Schematic of D4 mouse model and tamoxifen dosimetry schedule. (B, C) Representative flow cytometry plot (B) and quantitation of FDG reactive cell incidence (C) in TOP-D4 (red) or TOPgal (black) basal cells (Lectin^+^/EpCam^+^). (D, E) Representative H&E-stained, tamoxifen-treated TOPgal (D) and TOP-D4 (E) trachea sections. (F–I) Representative tamoxifen-treated TOPgal (F, H) and TOP-D4 (G, I) sections stained with antibodies directed against K14 (green, F, G), BrdU (red, F, G), CCSP (green, H, I) and acetylated tubulin (orange, H, I). (J–L) Quantitation of epithelial height (J), cellular phenotype (K), and BrdU incidence (L) in TOPgal (black) and TOP-D4 (red) tracheas. Scale bars = 100 µm; *p < 0.05, **p < 0.005

In order to assess whether β-catenin directs basal cell growth and lineage choice, TOPgal reporter mice were crossed with a K14-dependent, oestrogen-responsive and *N*-terminally truncated β-catenin transgenic mouse line (Δ*N*-β-cateninER, termed D4) [19]. These TOPgal × D4 (TOP-D4) bitransgenic mice activate β-catenin only in K14-expressing cells upon continuous tamoxifen exposure [35] (Figure 4A). We treated mice with 5 mg tamoxifen via intraperitoneal injection on days 0, 2, 4, 7 and 9 and culled the mice on day 10 (Figure 4A). As above, FDG flow cytometry was used to compare the abundance and phenotype of β-catenin–TCF signalling cells (Figure 4B). We observed increased β-catenin–TCF signalling, specifically within tracheal basal cells, only after tamoxifen treatment (Figure 4B, C). This tamoxifen-dependent increase was confirmed in TOP-D4 mice with whole-mount staining for β-galactosidase (see Supporting information, Figure S4).

The epithelial height and cellular density of tamoxifen-treated TOP-D4 tracheas was significantly increased relative to controls (Figure 4D, e; height quantitated in Figure 4J). Immunostaining also revealed that TOP-D4 tracheas contained significantly more basal cells and increased CCSP-expressing cell differentiation (Figure 4F–I, k). The lack of differences in ciliated cell abundance was most likely due to the long half-life of this cell type in undamaged airways (Figure 4H, I, k) [36]. We also observed significantly increased proliferation in tamoxifen-treated TOP-D4 tracheas relative to controls (Figure 4L). All of these results were verified in independent experiments by flow cytometry (data not shown). Thus, tracheal β-catenin–TCF signalling directs airway basal cell growth and differentiation.

### Basal cell β-catenin signalling induces a tracheal early EMT phenotype

**Figure 5:**
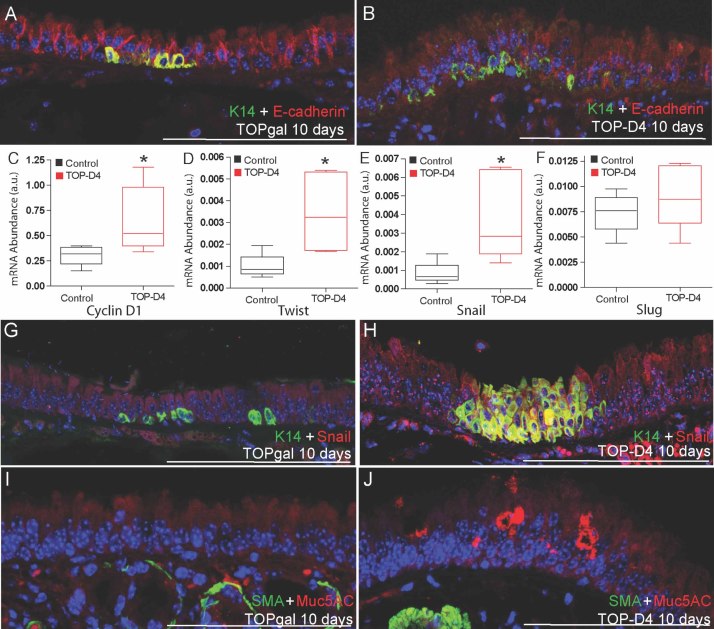
Tracheal basal cell β-catenin activation promotes early EMT phenotypes. (A, B) Representative tamoxifen-treated TOPgal (A) and TOP-D4 (B) sections stained with antibodies directed against K14 (green) and E-cadherin (red). (C–F) Quantitative PCR analysis of total RNA isolated from TOPgal (control, black) and TOP-D4 (red) tracheal epithelial cells following tamoxifen treatment. Relative Cyclin D1 (C), Twist (D), Snail (E) and Slug (F) abundance was assessed and normalized to β2-microglobulin, using 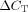 calculations. (G–J) Tamoxifen-treated TOPgal (G, I) and TOP-D4 tracheal sections were also stained with antibodies directed against K14 (green, G, H), Snail (red, G, H), smooth muscle actin (SMA, green, I, J) and Muc5AC (red, I, J). Graphs (C–F) are box-with-whisker plots; scale bars = 100 µm; asterisks *p < 0.05

Previous studies have established that β-catenin regulates epithelial fate via modulation of cellular EMT phenotypes [18]. To determine whether β-catenin activation induced an EMT within the trachea, we examined E-cadherin abundance and β-catenin target gene expression in WT and TOP-D4 tracheas. We found that tamoxifen-treated TOP-D4 tracheas exhibited reduced E-cadherin levels throughout their epithelium relative to controls (Figure 5A, b). Quantitative image analysis revealed that these differences were statistically significant (control average pixel intensity 141.7 ± 11.1 out of 256 versus 97.3 ± 8.2 in TOP-D4).

We next isolated RNA from five control and five TOP-D4 tamoxifen-treated tracheas and examined the expression of EMT-associated β-catenin target genes Twist, Snail, Slug and Cyclin D1. TOP-D4 samples exhibited significantly increased expression of canonical Wnt target genes Cyclin D1, Twist and Snail relative to controls (Figure 5C–F). We also saw an increase in MMP3 and MMP7 expression in TOP-D4 tamoxifen-treated tracheas (see Supporting information, Figure S5). Dual fluorescent immunostaining confirmed that transgene activation increased Snail expression (red, Figure 5G, h). We assessed epithelial smooth muscle actin and Muc5AC cell abundance to determine whether β-catenin induced an EMT (Figure 5I, j). We found that although β-catenin activation increased Muc5AC^+^ goblet cell abundance, it did not promote tracheal epithelial smooth muscle actin expression. This indicates that tracheal β-catenin activation only promotes a partial or early EMT.

### *In vitro* E-cadherin inhibition phenocopies β-catenin activation

**Figure 6:**
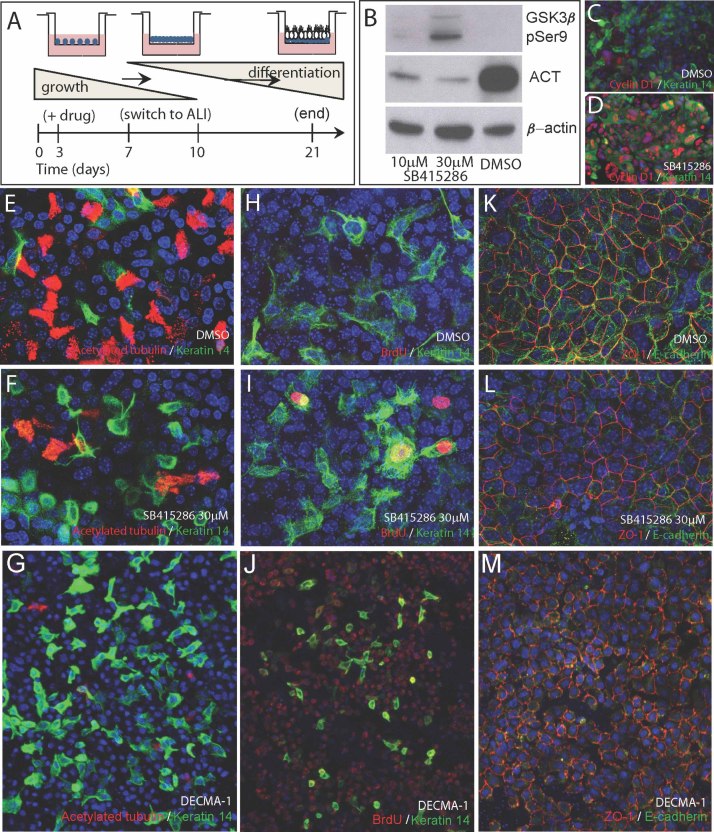
E-cadherin inhibition phenocopies *in vitro* tracheal epithelial β-catenin activation. (A) Schematic of murine tracheal epithelial cell (MTEC) air–liquid interface (ALI) model. (B) Protein blot of DMSO, 10 and 30 µM SB415286-treated ALI cultures using antibodies directed against GSK3β phospho-Serine9, acetylated tubulin (ACT) and β-actin. (C–M) Wholemount antibody staining of DMSO (C, E, H, K), 30 µM SB415286 (D, F, I, L) and DECMA-1 treated (G, J, M) ALI cultures. Antibodies included Keratin 14 (green, C–J), Cyclin D1 (red, C, D), ACT (red, E, F), BrdU (red, H–J), ZO-1 (red, K–M) and E-cadherin (green, K–M). DAPI was used as a nuclear counterstain (C–M)

To assess whether β-catenin influenced tracheal epithelial phenotypes through direct modulation of intercellular adhesiveness, we used a well-established air–liquid interface (ALI) model (Figure 6A) [10]. Murine ALI cultures were grown in the presence of the GSK3β-inhibitory (β-catenin-activating) drug SB415286, or the E-cadherin inhibitory monoclonal antibody DECMA-1; DMSO was used as a control [37–39]. Protein analysis of cell lysates harvested at day 21 revealed that treatment with either 10 or 30 µM SB415286 increased GSK3β Serine 9 protein phosphorylation (Figure 6B). We also observed increased expression of the canonical Wnt target protein Cyclin D1 upon treatment with 30 µM SB415286 (Figure 6C, D).

Interestingly, we found that although SB415286-, DECMA-1- and DMSO-treated cells appeared similar while in submersion culture, only DMSO-treated cells exhibited abundant acetylated tubulin differentiation following the switch to an air interface (red, Figure 6E–G). Western blots of protein isolated from these cultures confirmed this result (Figure 3B). Treatment with SB415286 and DECMA-1 also moderately increased K14-expressing basal cell abundance (green, Figure 6E–G). Separately, we assessed cell proliferation following treatment with SB415286 or DECMA-1 by examining BrdU nucleoside incorporation. We found that both β-catenin activation and direct E-cadherin inhibition increased epithelial cell proliferation relative to controls (Figure 6H–J). Additionally, although all cultures initially achieved confluence and exhibited liquid impermeability, DECMA-1 administration perturbed this air–liquid interface within 10 days. We therefore examined both cell surface E-cadherin (green, Figure 6K–M) and ZO-1 protein abundance (red, Figure 6K–M) in ALI cultures treated with SB415286, DECMA-1 and DMSO. Intercellular E-cadherin staining was reduced but there were similar ZO-1 levels in confluent ALI membranes treated with both SB415286 and DECMA-1. These results suggest that E-cadherin-dependent intercellular adhesions may themselves be responsible for determining cellular β-catenin signalling activity and subsequent tracheal epithelial growth and differentiation.

### β-Catenin signalling directs human airway epithelial growth and adhesiveness

**Figure 7:**
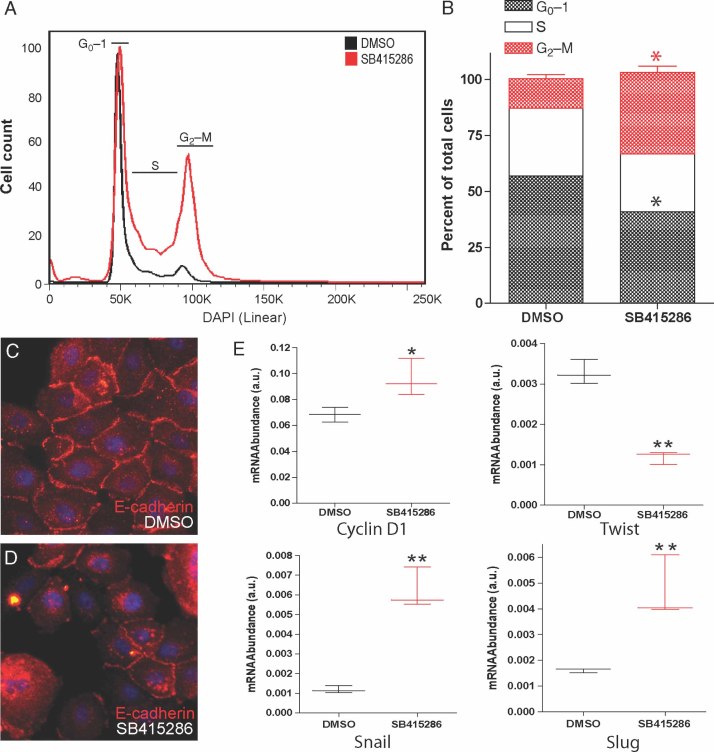
Canonical Wnt signalling promotes human airway cell proliferation and EMT-like phenotypes. (A, B) Representative flow cytometry plot (A) and quantitation (B) of immortalized human bronchial epithelial cell (iHBEC) cell cycle phase following exposure to DMSO (black line, A) or 30 µM SB415286 (red line, A). (C, D) E-cadherin antibody staining (red) of iHBECs treated for 24 h with DMSO (C) or 30 µm SB415286 (D). (E) Quantitative PCR analysis of total RNA isolated from iHBECs treated for 24 h with DMSO (black) or 30 µM SB415286 (red). Cyclin D1, Twist, Snail and Slug transcript abundance was normalized to 18S and quantitated using 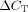 calculations. Graphs (E) represent box-with-whisker plots; *p < 0.05, **p < 0.005

To determine the relevance of β-catenin signalling in human airways, we treated immortalized human bronchial epithelial cells (iHBECs) with the β-catenin-activating drug SB415286 (30 µM). iHBECs express keratins 5 and 14, form tight intercellular adhesions and exhibit multipotent differentiation when cultured at an air–liquid interface [40]. Flow-cytometric analysis of SB415286-treated iHBECs revealed significantly reduced G_0_–1- and increased G_2_–M-phase cells relative to DMSO-treated controls (Figure 7A, b). This was accompanied by reduced cell-surface E-cadherin protein abundance (Figure 7C, D), as well as up-regulation of EMT-associated β-catenin target genes, including Cyclin D1, Snail and Slug (Figure 7E).

## Discussion

In this study, we report that β-catenin regulates tracheal epithelial basal cell growth and lineage choice. We demonstrate that β-catenin becomes activated within basal cells subsequent to tracheal damage and that inhibition of β-catenin signalling decreases cell proliferation and inhibits normal epithelial repair. Separately, K14-positive, basal cell-specific β-catenin–TCF activation restricts ciliated cell differentiation, promotes increased proliferation and reduces cell adhesiveness, both *in vitro* and *in vivo*. In humans with basal cell-derived preinvasive squamous lung cancer lesions, we observed positive correlations between elevated β-catenin signalling, disease severity, epithelial proliferation and an EMT phenotype. All of these effects were recapitulated following direct inhibition of E-cadherin, supporting a mechanism in which progenitor cell β-catenin signalling may direct airway epithelial growth and lineage choice via modulation of intercellular adhesiveness.

The results of this study are consistent with earlier studies investigating the role of β-catenin in distal lung development, homeostasis and repair. In particular, genetically modified mouse models in which β-catenin is activated throughout developing airways have shown reduced ciliated cell differentiation, expansion of bronchio-alveolar duct junction stem cells (BASCs) and increased epithelial proliferation [13–17, 41]. Some of these models also demonstrate spontaneous lung tumour development and hyperplastic epithelial polyp formation that is reminiscent of human lung cancer [14, 15].

In addition to these similarities, there were several distinctions between our results and those of earlier studies. Specifically, the current study is the first to demonstrate a direct relationship between basal cell β-catenin, intercellular adhesiveness, epithelial growth and lineage choice in upper airway progenitor cells. We found that β-catenin–TCF signalling within tracheal basal cells was by itself sufficient to promote epithelial proliferation, enhanced basal and secretory cell differentiation, and hyperplasia. We also found that β-catenin signalling within tracheal basal cells (including a stem cell subset) was necessary for normal epithelial repair. Importantly, the present work represents an investigation of the role of β-catenin signalling specifically within K14-expressing basal cells of adult mice upon exposure to exogenously administered tamoxifen. The mouse models used in our study therefore avoid any potentially confounding developmental, compensatory and/or off-target transgene effects that have been a component of several earlier studies [13–16].

In this study we found that β-catenin directly modulates early cellular EMT phenotypes, including E-cadherin abundance and transcription of Twist, Snail, Slug and Cyclin D1. We determined that E-cadherin inhibition and subsequent loss of adherens junctions may function as a molecular switch to regulate tracheal epithelial cell growth and differentiation. These results are consistent with identified roles for both β-catenin and E-cadherin in directing epithelial cell phenotypes in other tissues [18]. Notably, skin development and tumour initiation are both known to be regulated by basal stem cell β-catenin signalling influencing intercellular adhesions [20, 42–44]. Based on these results, we propose that cadherin—catenin crosstalk may modulate airway progenitor cell growth, differentiation and adhesiveness.

Although associations between pre-invasive cancer severity and early cellular EMT phenotypes already exist, the mechanisms responsible for driving these lung tumour initiation and progression have largely remained elusive [3]. It is now appreciated that dysregulated β-catenin–TCF signalling is of central importance in promoting EMT phenotypes associated with epithelial diseases [18]. The present study provides the first evidence that increased β-catenin–TCF signalling could directly influence lung EMT phenotypes, at least partially, through modulation of cellular adhesions. Our results also provide a simple potential mechanism to explain recent observations that increased canonical Wnt signalling is associated with poor human lung cancer outcomes [45]. Finally, these results may provide additional explanations as to how pulmonary Snail over-expression may by itself promote lung tumour development in animal models [46].

The functional links we identified between β-catenin signalling, E-cadherin inhibition via Twist, Slug and Snail, and airway basal cell growth and differentiation may explain how canonical Wnt signalling pathway components become over-expressed in squamous cell carcinomas, despite a paucity of identified β-catenin mutations in this disease [47]. Overall, our study identifies novel roles for β-catenin in normal and disease-associated tracheal progenitor cell growth and lineage choice. These results increase our understanding of airway epithelial cell biology and identify a potentially important regulatory mechanism in human lung cancer initiation.

## SUPPORTING INFORMATION ON THE INTERNET

The following supporting information may be found in the online version of this article:

**Figure S1.** Flow cytometry plots of TOPgal tracheal epithelial cell preparations.

**Figure S2.** Flow cytometry analysis of TOPgal positive and negative tracheal epithelial cell proliferation.

**Figure S3.** Submucosal gland development and homeostasis are unaffected by β-catenin inhibition.

**Figure S4.** Wholemount X-gal staining of β-catenin–TCF activity.

**Figure S5.** Enhanced MMP transcription accompanies tracheal basal cell β-catenin signalling.
