# Supplementary figures and images for: β-Catenin determines upper airway progenitor cell fate and preinvasive squamous lung cancer progression by modulating epithelial–mesenchymal transition
Source: J Pathol. 2012 Jan 17;226(4):575–87. doi: 10.1002/path.3962 (PMC3434372; doi:10.1002/path.3962)

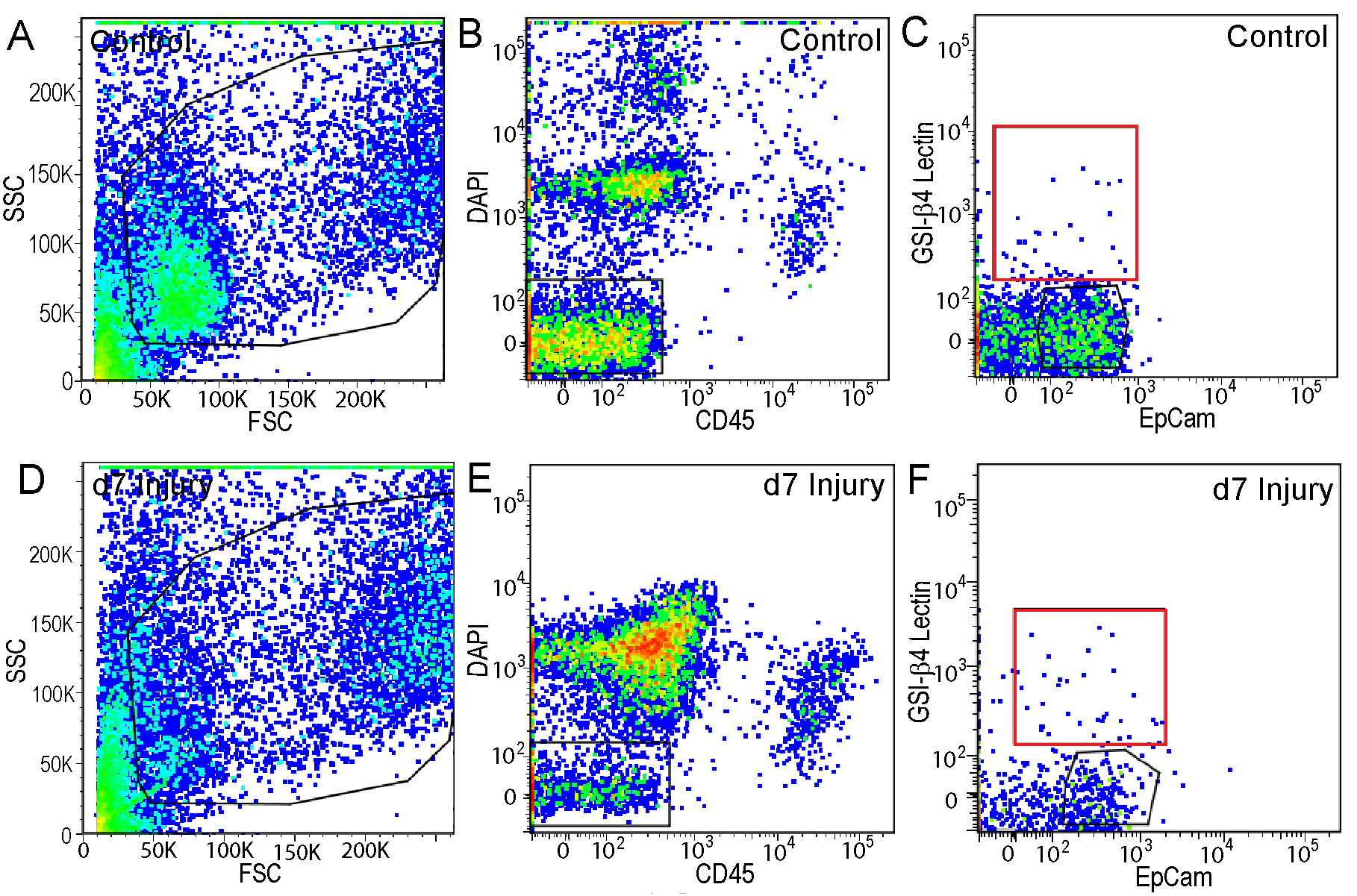

Supplement: Supplementary file 1 [file path0226-0575-SD1.tif]

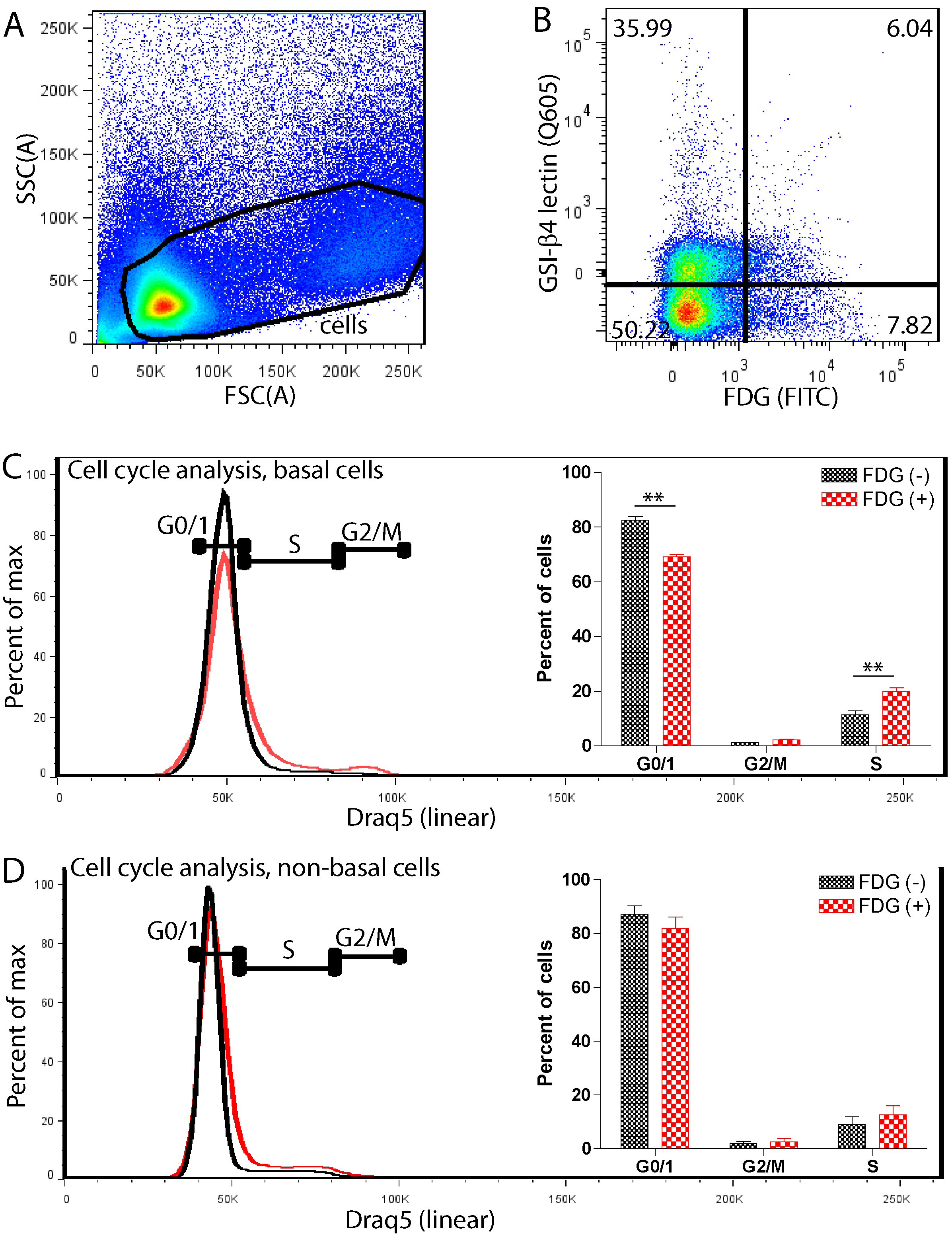

Supplement: Supplementary file 2 [file path0226-0575-SD2.tif]

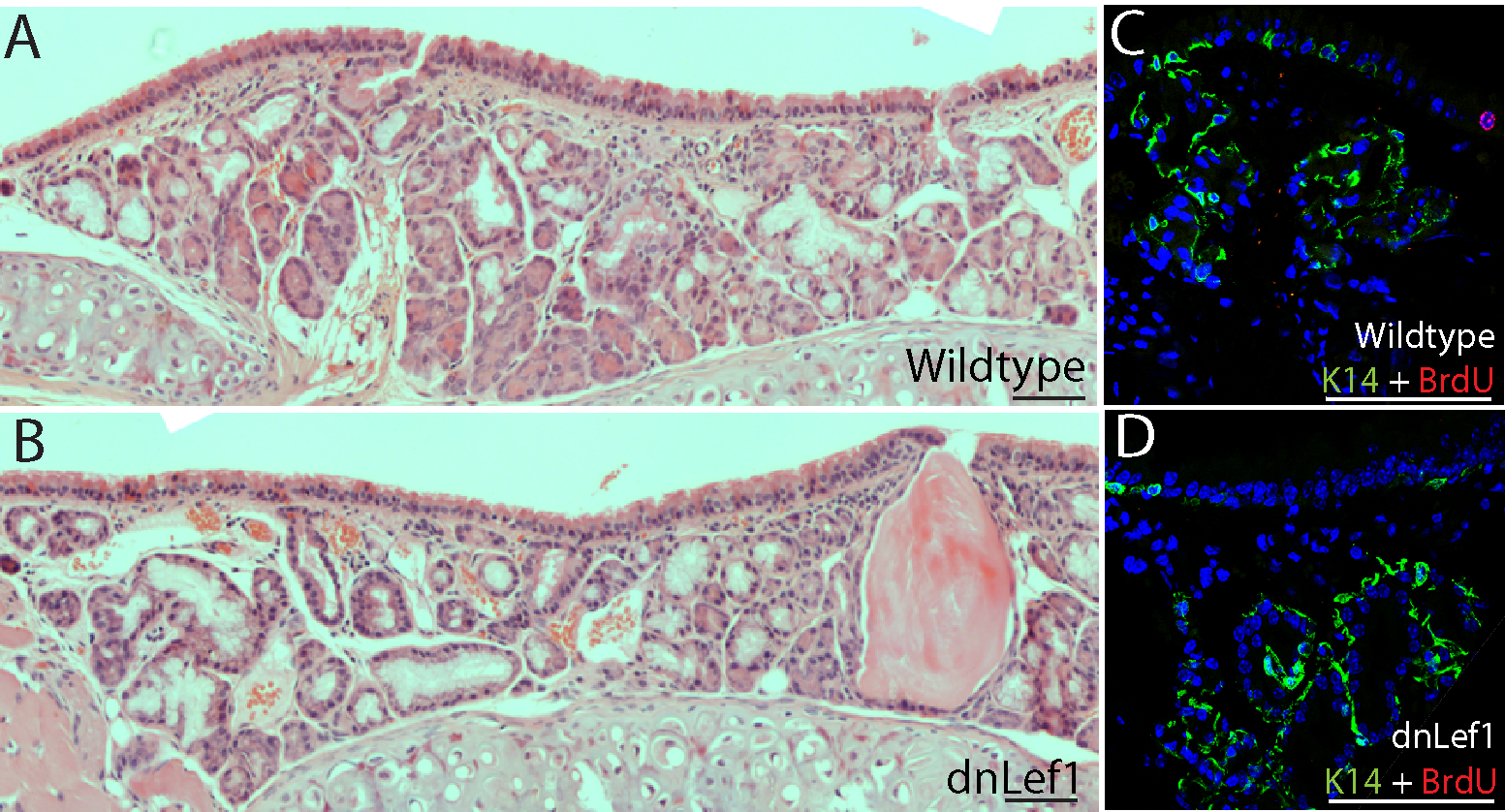

Supplement: Supplementary file 3 [file path0226-0575-SD3.tif]

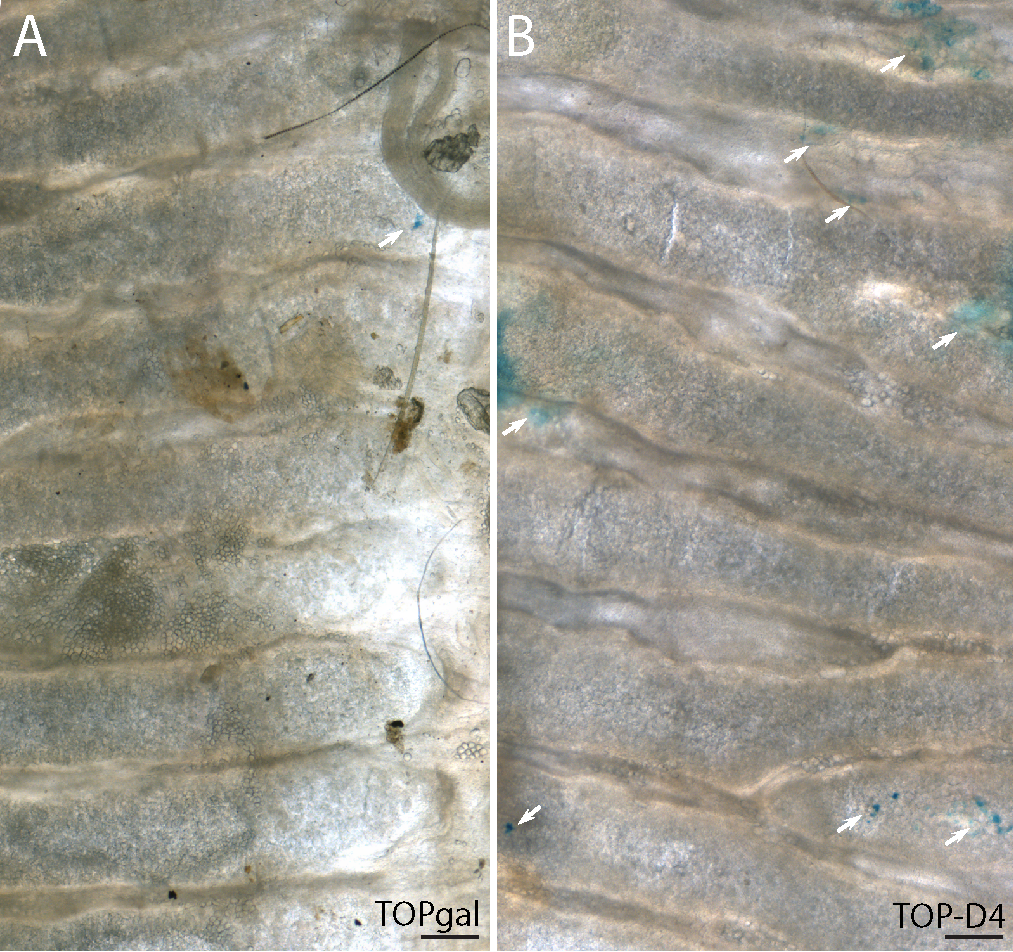

Supplement: Supplementary file 4 [file path0226-0575-SD4.tif]

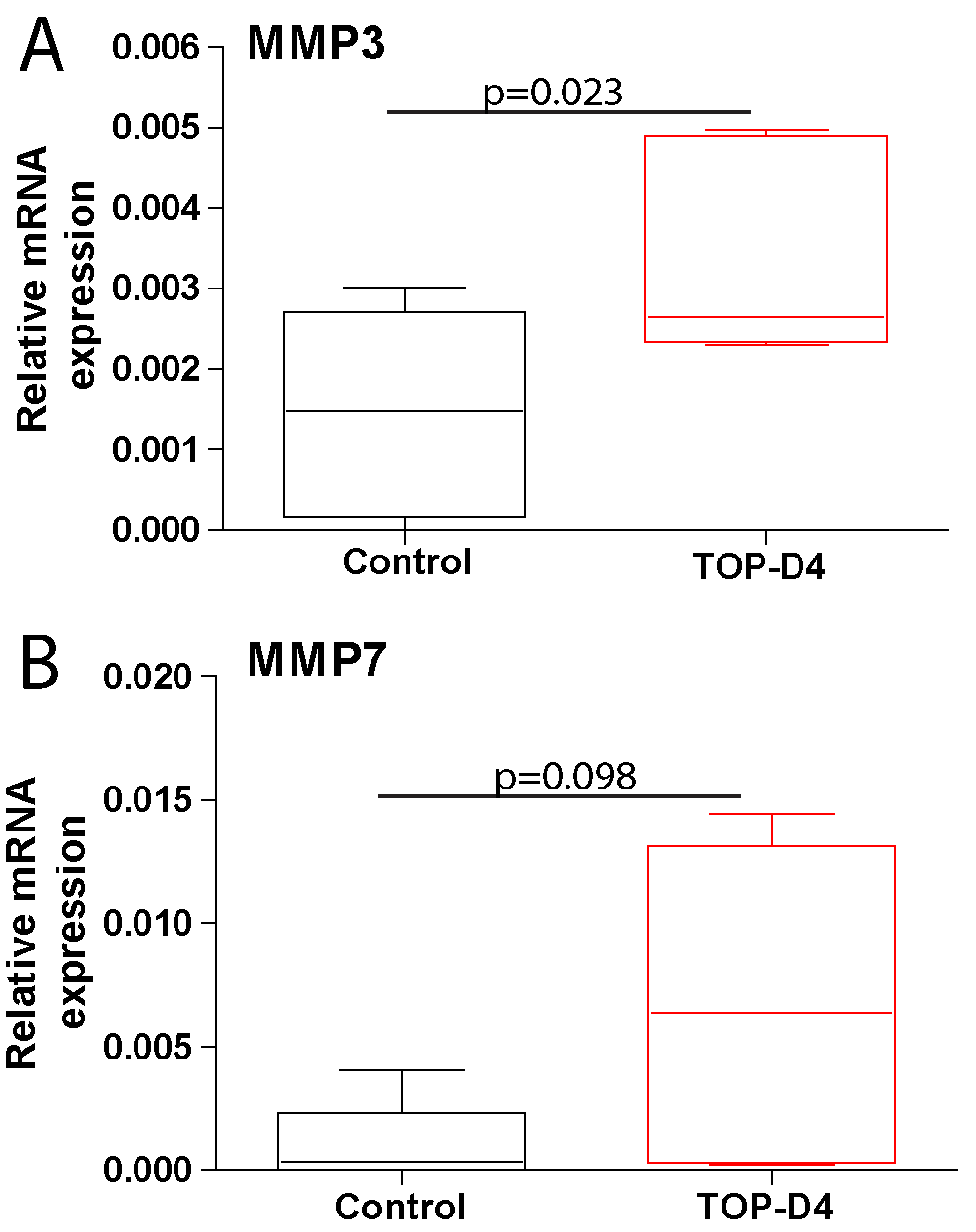

Supplement: Supplementary file 5 [file path0226-0575-SD5.tif]
